# The differences of enamel hardness post application of silver diamine fluoride and fluoride varnish on demineralized tooth surface (in vitro)

**DOI:** 10.1038/s41598-025-31343-0

**Published:** 2025-12-17

**Authors:** Irmaleny Irmaleny, Hendra Dian Adhita Dharsono, Nasya Salsabil Ash-shafa

**Affiliations:** 1 Department of Conservative Dentistry, Faculty of Dentistry, Universitas Padjadjaran, Jawa Barat, Bandung, 40132 Indonesia; 2 Undergraduate Program of Faculty of Dentistry, Universitas Padjadjaran, Bandung, Indonesia

**Keywords:** Enamel hardness, Silver diamine fluoride, Fluoride varnish, Remineralization, Health care, Materials science, Medical research

## Abstract

This research aimed to determine the difference in enamel hardness after the application of silver diamine fluoride and fluoride varnish on the surface of demineralized tooth. An in vitro true experimental research was conducted by collecting 27 maxillary premolars, which were separated into crowns and roots and divided into three groups (control, silver diamine fluoride, fluoride varnish). Demineralization was performed on all groups. Silver diamine fluoride remineralizing agent and fluoride varnish were applied for 3 min to the tooth enamel surface before the specimens were immersed in artificial saliva and placed in an incubator at 37 °C for 7 days. Tooth enamel hardness was measured using the MicroVickers hardness tester before demineralization, after demineralization, and after remineralization. The data analysis used in this research was one-way ANOVA with post hoc Tukey test for analyzing the differences in enamel hardness between groups and paired t-tests for analyzing the differences in enamel hardness in each group. The level of significance *α* was set at ≤ 0.05. All groups showed a significant increase in enamel hardness after remineralization (*p* < 0.05), with the highest mean value observed in the silver diamine fluoride group. Post hoc Tukey analysis revealed a significant difference between silver diamine fluoride and fluoride varnish groups (*p* < 0.05). There was a significant difference in enamel hardness between silver diamine fluoride and fluoride varnish post-application on the demineralized tooth surface. Silver diamine fluoride may be more effective option in managing early enamel demineralization and preventing the progression of caries.

## Introduction

Enamel is the hardest substance in the human body, comprised of hydroxyapatite crystals as the most dominant mineral element, and it has the protective role for deeper teeth structures^1,2^. The high amount of mineral content builds up the strength and hardness of the teeth^3^. Hardness is one of the substantial physical characteristics of the teeth layer, which depicts the teeth ability to withstand persevering plastic deformation and indentation^4^.

Dental caries has been permanently established as the disease with the highest prevalence, as stated in a study by Global Burden of Disease (GBD) 2017^5^. According to the 2018 Basic Health Research (Riset Kesehatan Dasar [Riskesdas]), 88.8% of Indonesians suffered dental caries, indicating an increased prevalence compared with the previous survey year^6^. The imbalance in the dynamic process of enamel demineralization and remineralization is the etiology of dental caries^1^.

Enamel demineralization occurs when the pH of the solution surrounding the enamel surface is below 5.5^3^. The constant demineralization will cause the vanishing of the enamel prism, resulting in the formation of microporous in the enamel layer^7^. The presence of porous causes the decline of enamel hardness^3^. Demineralization will discontinue if the acid concentration decreases and calcium or phosphate concentration increases in the saliva, indicating the start of remineralization^8^. Calcium and phosphate ion are re-deposited on the crystal porous in the demineralized teeth structure. Remineralization is a natural restoration process that occurs at a slow pace and is insufficient to protect an individual against demineralization without support from supplementary materials to increase the remineralization effect^8,9^.

The role of fluor as an effective remineralization material has been recognized for a long time and has been long-termed tested^10^. Silver diamine fluoride (SDF) and fluoride varnish are the fluoride-based remineralization materials that can be the options for caries lesion treatment at different stages of caries development due to their characteristics, i.e., non-invasive, effective, non-harmful, and comfortable to the patients with various age^11^. SDF can concurrently cease caries development and prevent the development of new caries, resulting from the combination effects produced by its silver and fluoride contents^12^. SDF effectively stops the development cavity caries lesion without pulp involvement, prevents caries lesion in exposed root surfaces, and treats teeth hypersensitivity^11^. The major side effect of SDF application is blackening discoloration of the tooth surface, impairs tooth aesthetics^13^.

Fluoride varnish has been widely used to prevent new caries lesions on healthy teeth, management of existing non-cavited caries lesion in patients with moderate to high caries risk, and treat tooth hypersensitivity^11,14,15^. Fluoride varnish prolongs contact time between fluoride varnish and enamel surface; hence, greater uptake of fluoride in enamel^16^.

The characteristic measurement of the teeth surface using a microhardness test is a common method utilized to investigate the change of enamel surface following the remineralization and demineralization cycle, especially with the Vickers method^17,18^. Some in vitro studies have compared the potential of SDF and fluoride varnish as remineralization materials. However, there were diverse results from each study. Based on a study by Farhadian et al., SDF had a higher remineralization potential compared to fluoride varnish^19^. A study by Mohammadi et al. mentioned that SDF and fluoride varnish yielded similar results in preventing demineralization^17^. Another diverse statement was made by Akyildiz et al., which stated that fluoride varnish was discovered to be more effective in the early enamel lesion remineralization compared to SDF^20^. This research aimed to evaluate the effects of silver diamine fluoride and fluoride varnish on the enamel hardness of demineralized tooth surfaces.

## Materials and methods

This research used the true experimental research design and was conducted in vitro. This research has acquired approval from the Research Ethical Committee of Universitas Padjadjaran (662/UN6.KEP/EC/2023) and approval from the Dean of Faculty of Dentistry Universitas Padjadjaran (4343/UN6.F.1/ PT.01.04/2023). All methods were performed in accordance with the relevant guidelines and regulations, including the Declaration of Helsinki. Informed consent was obtained from all the subjects. This research was conducted in May 2023 at the Laboratory of Metals and Crystallography of Faculty of Manufacturing Technology of Universitas Jenderal Achmad Yani and the Microbiology Laboratory of Faculty of Dentistry of Universitas Padjadjaran. The research specimens were 27 maxillary premolars, as determined by the Federer Formula ((n-1)(k-1) ≧ 15)^21^. The specimens criteria in this research were extracted teeth without restorative material, caries, and fractures, and teeth with fully-formed roots. The condition of each tooth was verified by visual inspection under magnification.

### Specimens preparation

All specimens—27 maxillary premolars—were cleaned and disinfected in 5.25% NaOCl solution for 5 min, followed by rinsing with distilled water^8^. The specimens were then stored in 0.9% saline solution at room temperature (20–25^°^C) until further experimental procedures were performed^8^. The crown portion of the teeth was removed horizontally from the root portion 1 mm below the cementoenamel junction (CEJ) using a carborundum disc. The crown portion of the teeth was buried in a transparent resin mold sized 3 × 3 × 2 cm with the buccal surface of the crown facing upwards^8^. The enamel surfaces of all specimens were flattened and smoothed using a silicon carbide paper grit sized 320, 600, and 1200, respectively^20^. Each specimens was numbered 1–27 behind the resin mold.

### Research procedure

Each specimens was measured for its initial enamel hardness using the MicroVickers Hardness Tester *(Metkon Duroline-M*,* Turkey)* with 200 gram loads for 10 s on three indentation points distancing ± 0.5 mm vertically^19^. Each specimens enamel hardness measurement results were based on the mean value from 3 indentation points with Vickers Hardness Number (VHN) units or kg/mm^2^.

After the initial enamel hardness measurement, the specimens were demineralized by soaking them in an isotonic drink with a pH of 4 for 30 min^8^. Demineralization was conducted to stimulate artificial carious lesions to determine remineralization efficiency^20^. The specimens were rinsed with distilled water and dried using a chip blower. The enamel hardness was re-measured using the MicroVickers Hardness Tester to obtain the enamel hardness number post-demineralization.

The specimens were randomly divided into three groups: group I as the control group without any interventions, group II as the treatment group with the application of 30% SDF *(Cariestop*,* Biodinamica*,* Brazil)*, and group III as the treatment group with the application of 5% NaF White Varnish *(Clinpro™ White Varnish*,* 3 M ESPE*,* USA)*. All specimens in group I was soaked in artificial saliva following the AFNOR method, containing NaCl 12 mM, KSCN 3.4 mM, NaHCO_3_ 17.8 mM, KCl 16 mM, Urea 22 mM, NaH_2_PO_4_ 1.5 mM, KH_2_PO_4_ 1.5 mM, and HCl dissolved in distilled water, and kept without any interventions^8^. All specimens in group II were treated with 30% silver diamine fluoride (SDF) solution applied to the buccal crown surface for 3 min, followed by gentle removal of excess material using a cotton roll and subsequent immersion in artificial saliva. Specimens in group III were treated 5% sodium fluoride (NaF) White Varnish applied to the the buccal crown surface for 3 min, followed by gentle removal of excess material using a cotton roll and subsequent immersion in artificial saliva^19^.

All specimens were soaked in artificial saliva and stored in an incubator at 37 °C for seven days; the artificial saliva was replaced daily^20^. After seven days, all specimens were washed using sterile distilled water and dried using a chip blower. The enamel hardness was re-measured using the MicroVickers Hardness Tester to measure tooth hardness following remineralization in the control and treatment groups. The measurement result data were statistically tested using the paired t-test, one-way ANOVA, and post hoc Tukey test in the IBM SPSS Statistics version 29 software.

## Results

**Fig. 1:**
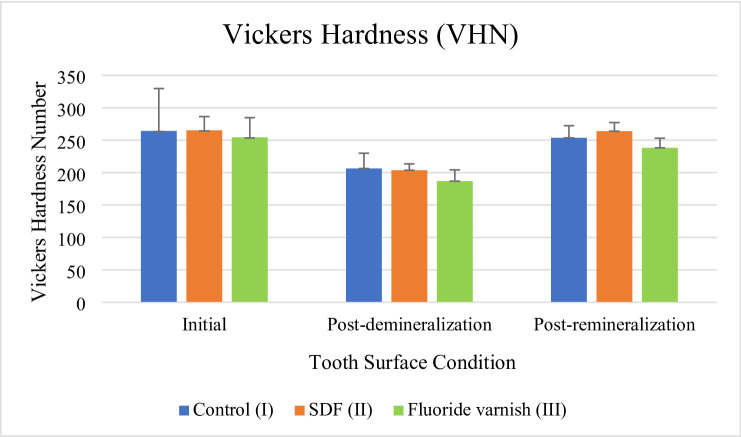
Mean result of initial, post-demineralization and post-remineralization enamel hardness measurement.

The measurement results data of the initial, post-demineralization, and post-remineralization hardness from 27 maxillary premolar specimens of each group can be seen in Fig. 1.

The mean values of the initial enamel hardness, as presented in Fig. 1, in groups I, II, and III were 264.29 ± 65.64 VHN, 265.33 ± 21.27 VHN, and 254.46 ± 30.40 VHN, respectively. The mean values of the post-demineralization enamel hardness in groups I, II, and III were 206.31 ± 23.64 VHN, 203.75 ± 9.80, and 186.90 ± 17.30 VHN, respectively. The mean scores of the post-remineralization enamel hardness in groups I, II, and III were 253.66 ± 18.71 VHN, 263.95 ± 13.32 VHN, and 238.19 ± 14.92 VHN, respectively.

The one-way ANOVA test results of all groups in the initial, post-demineralization, and post-remineralization are represented in Table 1.

**Table 1:** One-way ANOVA test on three groups.

Test Group	Enamel Hardness (VHN)					
	Initial		Demineralization		Remineralization	
	Mean	SD	Mean	SD	Mean	SD
Control	264.29	65.64	206.31	23.64	253.66	18.71
SDF	265.33	21.27	203.75	9.80	263.95	13.32
Fluoride varnish	254.46	30.40	186.90	17.30	238.19	14.92
*p*-value	0.844		0.061		0.007	

Based on the analysis results of the one-way ANOVA in Table 1, the significance value of the initial and post-demineralization enamel hardness of all groups was *p* > 0.05, indicating an insignificant difference in the initial and post-demineralization enamel hardness of group I, II, and III. The insignificant difference indicates that the results data of the initial hardness was homogenous, and the quantity of demineralization treatment in all groups was proportional.

The derivation values of the post-demineralization and post-remineralization enamel hardness of all groups are presented in Table 2.

**Table 2:** Paired t-test on three groups after demineralization and remineralization.

Test Group	Mean difference	SD	*p*-value
Control	-47.35	29.04	< 0.001
SDF	-60.19	15.04	< 0.001
Fluoride varnish	-51.28	23.40	< 0.001

The paired t-test results of all groups in Table 2 showed a significance value of *p* < 0.05, indicating a significant difference in the post-demineralization and post-remineralization enamel hardness. The mean value of enamel hardness increased after remineralization using SDF and fluoride varnish, particularly in group II (SDF application), which yielded the highest increase.

The one-way ANOVA results of the post-remineralization data in Table 1 showed a significance value of *p* < 0.05, indicating a significant difference in enamel hardness in all groups after applying SDF and fluoride varnish on the tooth surfaces. Hereafter, the paired post hoc Tukey test of the post-remineralization data was performed and presented in Table 3.

**Table 3:** Post hoc Tukey test.

Comparison		Mean difference	Std. Error	*p*-value
Test Group				
Control	SDF	-10.28	7.45	0.367
	Fluoride varnish	15.47	7.45	0.116
SDF	Control	10.28	7.45	0.367
	Fluoride varnish	25.75	7.45	0.006
Fluoride varnish	Control	-15.47	7.45	0.116
	SDF	-25.7	7.45	0.006

The post hoc Tukey test performed to compare between post-remineralization groups in pairs resulted in a significant difference in the enamel hardness between group II (SDF) and group III (fluoride varnish) (*p* < 0.05). However, there was an insignificant difference between group I (control) with group II (SDF) and III (fluoride varnish) (*p* > 0.05).

## Discussion

Teeth remineralization potential can be increased by the presence of additional extrinsic sources from calcium and phosphate ions^9^. This research results exhibited a significant difference in the enamel hardness post-demineralization and post-remineralization. The enamel hardness mean value increased after applying SDF and fluoride varnish and soaking in artificial saliva for seven days. The SDF group showed a higher increase in enamel hardness mean value (60.19 VHN) compared to the fluoride varnish group (51.28 VHN) and the control group (47.35 VHN). An identical finding was also discovered in the final enamel hardness mean value; the SDF group had the highest enamel hardness mean value and showed a significant difference with the fluoride varnish group.

In this research, the SDF and fluoride varnish group could exhibit remineralization effects in the demineralized specimens. The SDF group exhibited a higher enamel remineralization potential compared to the fluoride varnish and control groups. The high-concentrated fluoride in 30% SDF (35,400 ppm) promotes hydroxyapatite remineralization to generate the formation of silver phosphate and calcium fluoride, which acts as the fluoride reservoir during the cariogenic process^20,22^. Silver phosphate acts as an insoluble protective layer on the teeth surface, resulting in less calcium and phosphate ion loss on enamel^12^. The reaction between fluoride and hydroxyapatite initiates the gradual formation of fluorohydroxyapatite, which is more resistant to solubility^12^. The findings in this research are parallel with a study by Farhadian et al. which stated that the enamel hardness mean value in the SDF group was significantly higher than the fluoride varnish groups; therefore, the SDF fluor was concluded to have a greater effectivity^19^. Mohammadi et al., in their study, also stated a similar statement. The SDF-applied specimens group was more resistant to mineral loss compared to the fluoride varnish-applied group. However, a significant difference between both groups was not discovered^17^. A distinct statement was made by Akyildiz et al.; the NaF-applied specimens group yielded the highest enamel hardness and a more significant remineralization effect compared to the SDF group^20^.

SDF has been considered non-harmful, minimally invasive, and more practical in managing dental caries^13^. The increase of SDF concentration is discovered to increase the fluorohydroxyapatite contents^23^. The alkaline property of SDF is also favorable to the remineralization process. Moreover, SDF consists of silver ions, which act as the role of antibacterial^14^. The properties of SDF can affect the mineral content of the teeth, directly impacting the tooth hardness^12^.

The fluoride varnish group yielded a lower post-remineralization enamel hardness mean value compared to the SDF group. The increase of enamel hardness mean value post-demineralization and post-remineralization was mere than the SDF group. The fluoride intake by the tooth surface relies on its concentration and prolongation of contact time between fluoride and tooth structure^24^. The 5% fluoride varnish used in this research had a lower fluoride concentration (22,600 ppm) compared to SDF (35,400 ppm)^25^. The higher viscosity of fluoride varnish than SDF also enables the diffuse deceleration of fluoride ions into the enamel layer. In this research, the fluoride varnish was removed from the tooth surface after three minutes of application, and this limited contact time is presumed to have reduced the amount of fluoride available for enamel uptake^14^.

Saliva is fundamental in neutralizing acid and providing calcium and phosphate ions to support remineralization^26^. The artificial saliva in this research was utilized to replicate the remineralization condition in the oral cavity, which parallels with a suggestion from a study by Taofik et al., which mentioned the saliva composition following the AFNOR method^8^. This research results indicated an insignificant difference in the enamel hardness between the control and fluoride varnish groups. The control group yielded a higher mean value of final enamel hardness compared to the fluoride varnish group. This finding can be correlated with the artificial saliva composition used in this research. As mentioned by Taher et al. in their study, it verifies that artificial saliva containing potassium chloride, calcium chloride, and potassium dihydrogen phosphate can support enamel remineralization and increase hardness^27^. The mineral contents in artificial saliva are higher than in natural saliva, but it does not contain protein and enzymes. Therefore, the remineralization process can easily occur compared to the oral cavity^28^.

Some previous studies have reported the higher remineralization potential of SDF compared to fluoride varnish. However, the application of SDF generates a blackening discoloration on the teeth surface resulting from the oxidative property of silver ions on the silver phosphate layer^20,23^. The discoloration can be reduced by applying potassium iodide solution on the tooth surface after the treatment using SDF without decreasing SDF’s potential^19^.

The research limitations involve the difficulties of replicating the clinical oral cavity condition. The artificial saliva used in this research could not represent the complexity of the natural saliva. The tooth specimens used in this research was limited to only maxillary premolars, and the remineralization process occurred only in seven days. In addition, relatively small sample size also represents a limitation of this research. Future studies are recommended to include a larger sample size and utilize human natural saliva or conduct in vivo experiments to better simulate the clinical conditions.

## Conclusion

A significant difference was evaluate between enamel hardness post-application of SDF and fluoride varnish in demineralized tooth surfaces. The SDF application provided a higher enamel hardness compared to fluoride varnish. These findings may provide guidance in using SDF and fluoride varnish as preventive agents to improve enamel hardness in terms of remineralization process.

## Data Availability

The datasets used and/or analysed during the current research available from the corresponding author on reasonable request.
